# Enabling factors facilitating the use of neem-based remedies for the management of malaria in the Lower Shire District of Chikwawa, Malawi

**DOI:** 10.5281/zenodo.10887837

**Published:** 2014-10-02

**Authors:** Edson Dembo, Fraction Dzinjalamala, Annette Habluetzel

**Affiliations:** 1 School of Pharmacy, University of Camerino, Piazza dei Costanti, 62032 Camerino (MC), Italy; 2 Department of Pharmacy, College of Medicine, University of Malawi, Blantyre, Malawi

## Abstract

**Background:**

Malaria remains a major public health threat in Malawi, affecting mostly children under five and pregnant women. Despite the availability of chemotherapy and chemoprophylaxis, resistance to sulfadoxine pyrimethamine and the high cost and complicated regimen of artemether-lumefantrine have accelerated the use of home-based remedies for the management of malaria in Chikwawa district, Malawi. This study aimed to determine factors that facilitate the use of herbal remedies within communities in the management of malaria in the presence of free health care services, with the intention of assessing the feasibility of developing improved herbal products as anti-malarial prophylaxis.

**Materials and Methods:**

Data on factors driving the use of neem-based preparations commonly used in the management of malaria were collected through qualitative interviews and focus group discussions. Qualitative data were analysed drawing on the Framework Analysis approach.

**Results:**

Neem and moringa were identified as the principal plants used for the management of malaria, with neem being the most frequently used. Factors favouring the communal use of neem-based remedies included the habit of resorting to herbal remedies as first aid treatment, lack of drugs and proper medical care in modern health facilities, and the need for preventive anti-malarial remedies during the high-transmission season. The perceived effectiveness of neem-based herbal remedies was based on their fast action against the symptoms of malaria, thereby providing immediate relief to the patient, which might explain their wide-scale use for malaria treatment.

**Conclusions:**

Local communities prefer to use neem and/or moringa remedies for their primary healthcare needs in the management of malaria because of their ease of access, preparation and administration without frequent adverse events, as opposed to ACTs. These remedies are already being used as prophylaxis in unimproved/non-standardised formulation. This suggests that standardised herbal preparations would be culturally acceptable at community level. Evidence-based research is required to validate parasitological and clinical efficacy and determine safety of these anti-malarial herbs.

## 1 Introduction

Recent estimates suggest that, over the last decade, malaria -related mortality has been reduced by about 45% [1], thanks to an integrated approach including vector control, prompt diagnosis, and treatment of cases. The success of this decline is, in part, due to the wide-scale distribution of free or highly subsidised insecticide-treated bed nets (ITNs), indoor residual spraying (IRS) and artemisinin-based combination therapy (ACT). These malaria control strategies benefit individual users and, if implemented at sufficiently high coverage, confer significant benefits to the overall community through reduction of parasite transmission ([2,3];; reviewed in [4]). Despite this success, estimates by the Malawi Ministry of Health indicate that suspected malaria cases almost doubled from about 3.7 million cases in 2005 to 6.7 million cases in 2010 [5] and thereafter started to decline (to 4.9 million in 2012), which was probably due to increased and improved diagnosis as a result of the roll-out of malaria rapid diagnostic tests (mRDTs) (NMCP 2013;; unpublished data). Almost 50% of all suspected malaria cases occurred in children under the age of five. Malaria also constitutes about 40% of all hospitalisation of children under the age of five. Annually, malaria contributes at least 34% of all out-patient visits and 40% of all hospital deaths in the country [5-7] as well as accounting for 21-44% of the resources disbursed for health care [8].

In Malawi, chemotherapy and chemoprophylaxis are currently available for treating and preventing clinical episodes of malaria. However, there is an increasing spread of *Plasmodium falciparum* strains resistant to sulfadoxine pyrimethamine (SP), the anti-malarial drug which had been efficacious, safe, easily accessible and affordable during the last decade [9];; this has prompted a Malawi Government policy change of first-line treatment of malaria (reviewed in [5]) to artemether-lumefantrine (AL). AL, an artemisinin-based combination therapy (ACT), is ex-pensive if purchased commercially, is reportedly known to cause adverse events (such as nausea, headache and vomiting) and has limited use against malaria during pregnancy [10]. While SP is a single-dose regimen, AL is a six-dose regimen, with the second dose required to be taken immediately after 8 hours;; this complicated regimen makes compliance difficult, as has been reported elsewhere [11]. Taken together, this has generated serious problems for malaria prevention in pregnant women and for the management of malaria overall.

More than 60% of the health services in Malawi are government-owned and are provided for free at a point of delivery. In some areas, for instance in parts of Chikwawa such as Nchalo, where free government health facilities are not easily accessible, the population is served by non-profit private health organisations, mainly comprising Christian hospitals under the umbrella of the Christian Health Association of Malawi (CHAM), which provides services for very low user fees (highly subsidised by the government) in coordination with the government health service delivery policy. However, health sector funding is very poor and, as a result, health service delivery is impeded due to dilapidated buildings, non-functional equipment, lack of drugs and supplies as well as poorly motivated health workers [12]. Only half of the Malawian population lives within a 5-km radius of any kind of health facility. Although public health services are free for the patients, the cost of transportation to and from a facility deters individuals from accessing these free health services [12]. This, coupled with the fact that about 70% of Malawians lives in poverty [13], presents a major challenge when accessing paid-for health delivery services. Based on this background, communities in the district of Chikwawa tend to resort to home-based herbal remedies for the management of malaria.

In the Southern region of Malawi, particularly in the Lower Shire Districts of Chikwawa and Nsanje, local communities have been using jatropha (*Jatropha curcas* L., family *Euphorbiaceae*), neem (*Azadirachta indica* A. Juss., family *Meliaceae*) and moringa (*Moringa oleifera* Lam., family *Moringaceae*) for the management and prevention of malaria. The use of these herbal remedies might have accelerated over the years following the introduction of the Janeemo (Jatropha, neem, moringa) Project, funded by the Scottish Government International Development Fund, which is promoting the cultivation of these trees in Southern Malawi and in other parts of the country [14]. As a result, neem, moringa and jatropha trees are widespread in the Lower Shire Districts of Chikwawa and Nsanje. Extracts from different parts of these three tree species have been shown to have nutritional and medicinal proper-ties. In addition, these trees produce oil-rich seeds, which are exploited for the production of bio-fuels and soap at community level [14].

Despite being extensively used, there have been no clinical or field evaluation studies to validate the claimed anti-malarial properties of jatropha, neem and moringa plant extracts as prepared by local communities in Malawi. It is scientifically accepted that randomised controlled clinical trials are the gold standard to evaluate the efficacy and safety of drugs and remedies. Ethically, clinical trials cannot be conducted for unstandardised remedies despite their continued use. However, since malaria is a disease that people can recognise and self-diagnose at community level, we conducted a qualitative study to determine factors that facilitate community use of herbal remedies in the management of malaria in the presence of free health care services in Malawi. The intention was to assess whether such enabling factors confer the feasibility of developing an improved or standardised herbal product that can be used to reduce malaria transmission at community level.

## 2 Materials and Methods

### 2.1 Study area and population

This study was conducted in the rural areas of the Lower Shire District of Chikwawa in Malawi. The study area has a documented malaria incidence rate of 450 per 1000 per-sons per year in the general population, with a higher incidence rate of 730 per 1000 persons per year in children under the age of five [15]. The incidence tends to be high-er during the rainy season, between November and April. The study was conducted between March and July 2012 and enrolled members of the local community, both men and women, of varying age, socio-economic status, marital status/household-headship and literacy levels.

### 2.2 Selection of study communities

At lower governance level, Malawi is divided into Traditional Authority (TA) governance units [16]. In the district under study, four out of eight TAs were selected at random and the TA councils were consulted and briefed about the study. Once they had consented, the TA councils were asked to provide the names of the communities under their jurisdiction. These communities were pulled together and stratified based on access to free health care provided by government health facilities. Access to free government health facilities was defined as follows: One category of communities included those within the country’s recommended 5 km radius of government health facilities with-out any other geographical barriers. These were considered as communities with easy access to free health care. Communities with difficult access to free health care were de-fined as those outside a 5-km radius of government health facilities or those with geographical barriers to government health facilities regardless of geographical distance [16]. Communities were randomly selected from each stratum. A total of 247 individuals from four communities, two communities with easy access (Group Village Head-man (GVH) Misongwe and GVH Sande communities) and two communities with difficult access to free health care (GVH Sekeni 1 and GVH Sekeni 2), were enrolled in the study.

### 2.3 Data collection methods and selection of participants

This study employed two methods of qualitative data collection – focus group discussions (FGDs) and in-depth interviews (IDIs). For focus group discussions, groups of about 10 participants were invited with the help of the village’s knowledgeable person(s) or guide(s) in consultation with leaders of the local communities. With the help of two community guiders (one male and one female), appointed by the traditional leaders in each study community, a random selection of households was obtained from which individuals for FGDs were selected, balanced for age, gender and access to free health care services. In total, 24 FGDs were conducted, each lasting approximately one hour. The groups were asked about their perception of malaria as a health problem, how they manage the disease, perceived safety and efficacy of the treatment they seek, the prophylactic measures they apply, and perceived ad-vantages and disadvantages of the different treatments they seek for the management of malaria. They were also asked about the decision-making process in seeking health care for treatment and prevention.

IDIs were conducted to investigate key issues more deeply, such as the method of preparation of the mentioned medicinal plants and mode of administration;; the nature of herbal formulations as the communities may recommend as anti-malarial prophylaxis and other pertinent issues that arose in FGDs. The use of these two methods also helped in triangulation of responses between male and female respondents and allowed further clarification of salient issues. For IDIs, participants were re-sampled from previously conducted FGDs;; these were purposively sampled based on their breadth of knowledge of using herbal remedies in the communities under study. Saturation of ideas was reached after interviewing 40 participants.

Both FGDs and IDIs were facilitated by the corresponding author, who has considerable experience in malaria research in Malawi and in planning and execution of qualitative enquiries. During each inquiry, two experienced qualitative researchers (male and female) assisted in taking notes, which were used for consensus building where necessary, particularly where words of hidden meanings were used or in analysing the focus group inter-actions.

### 2.4 Data analysis

All interviews were conducted using the national language (Chichewa); the corresponding author and the two re-search assistants are fluent in both English (Malawi’s official language) and Chichewa. Transcription of the recorded information was carried out in English by the corresponding author and one of the two research assistants. Where differences emerged a third transcription was obtained from the second research assistant.

Information recorded on tape was transcribed and analysed inductively following a thematic analysis approach to identify, analyse and report the main themes. The qualitative data were analysed drawing on the Framework Analysis approach [17]. This approach involved familiarisation of the data, identification of the thematic frame-work, indexing, charting, mapping and interpretation of the data. The Framework Analysis approach has been developed to facilitate identification of community-based recommendations in the context of applied policy research [18], as was the case in this study.

### 2.5 Ethical considerations

Ethical approval was obtained from the College of Medicine Research and Ethics Committee (COMREC, P.02/12/1083). The interviewee(s) were informed of the study objectives, methods to be used for collecting data (tape recording), the voluntary nature to participate and their right(s) to withdraw from participation at any time should they feel uncomfortable with the study. Written informed consent was obtained before the qualitative enquiry began.

## 3 Results

### 3.1 Respondents of the study communities

A total of 247 individuals from four communities of the Lower Shire District of Chikwawa (Malawi), balanced for gender were involved in the study (Table 1). About half of the respondents were from areas where accessibility to free health care was considered difficult.

**Table 1.** Characteristics of FGD and IDI respondents by age, sex and access to free health care.

Description	Number	%
Number of FGDs	24	
Total respondents in FGDs	247	
Number of respondents for IDI	40	
Number of communities	4	
*Age of IDI respondents (years)*		
<25	5	13
25–34	10	25
35–44	9	22
45–55	7	18
>55	9	22
*Gender*		
Male	20	50
Female	20	50
*Access to free health care*		
Easy access	20	50
Difficult access	20	50

“*Malaria sickness derails household chores and developments, thereby affecting the socio-economic status of the household” (Man, FGD, GVH Sekeni 1)*

Regarding decision making for the health care problems within the family, most of the respondents reported that the decisions are taken on the basis of consultation between the spouses. However, it was noted that usually children spend most of the time with their mothers, as fathers are more frequently out of the home being involved in the productive chores necessary for the family welfare. Because of this, women more frequently make decisions about family health care needs. Men, however, are culturally considered as heads of households and felt they are the ones who make final decisions. When this was triangulated using FGD with men it was reported that women make decisions especially in the absence of their husbands.

All the respondents showed adequate knowledge of the seriousness of malaria infections. They knew that malaria is fatal and also recognised that the disease exacts a high economic toll on households.

### 3.2 Anti-malarial treatment behaviour

Generally, it was reported that the first-line treatment for malaria was effected at household level and consisted in the oral administration of antipyretics and analgesics. This was either by using leftover drugs from previous treatments sought from medical facilities or drugs that were bought in local shops.

“*When malaria is suspected, the first thing I do is to buy Panadol and Brufen. I administer this treatment to the ill child and observe for any improvement. If no change is observed then I start administering our home-based remedies. But if I see that it is taking longer and the child’s condition is not improving then I take the patient straight to the hospital for diagnosis and treatment”. (Woman, FGD;; GVH Misongwe)*

It was expected that anti-malarial treatment-seeking behaviour would be differentiated by the geographical accessibility to government health facilities. On the contrary, our data showed that there was a general trend of providing herbal remedies as first aid treatment to suspected malaria patients before seeking professional medical care. However, this choice was motivated by the erratic supply of anti-malarial drugs in free government health facilities and private health facilities as well as the service charges imposed for diagnosis and treatment in private health facilities.

From the free government health care facilities it was reported that sometimes patients and guardians have to queue for several hours before consultation and diagnosis. When the patient is confirmed to have malaria he or she may still be sent back home untreated due to unavailability of anti-malarial drugs. These health service delivery prob-lems were reportedly causing relevant loss of time to the families that rely mostly on subsistence farming. This time loss was presumed avoidable when readily available home -based herbal remedies were administered to the patient first.

“*We always think of going to the hospital, but the problem is that you see your friend coming back without being treated because of a lack of drugs at the government hospital after being in the queue for about 4 hours. So, you think about it and say, why should I go and waste time at the hospital when I have readily available treatment in the community?” (Man, FGD;; GVH Misongwe)*

Perceived lack of appropriate health care service was also influencing the treatment choices that were adopted. For instance, insufficient supply of child-friendly AL formulations necessitated caregivers to resort to anti-malarial herbal remedies.

“*Sometimes we use herbal remedies because we haven’t been properly assisted at the hospital. For example, that one [pointing at a 3-year old boy playing] is my grandson. Three days ago, he fell ill. We (my wife and me) took him to the hospital and he was diagnosed with malaria in their laboratory. But the doctor told us that they did not have the regimen suitable for his age. We just returned home and prepared neem-based remedy by boiling and gave it to him. See him now, he is happily playing. He is fully cured and we would not need to go back to the hospital”. (Man, GVH Sekeni 1)*

Experienced cases of treatment failure after oral administration of AL may also have catalysed caregivers’ preferences for herbal remedies as first line anti-malarial treatment.

“*I have a 4-year old child in my house. When he is ill of malaria and AL is administered his condition does not change no matter how many times he is given the appropriate dosage. But his body responds well when neem-based remedies are administered orally. When neem is administered orally, morning, afternoon and evening the same day the child gets well.” (Woman, FGD;; GVH Sande).*

Despite these challenges, respondents recognised the importance of seeking modern health care for the management of malaria and other illnesses. This was particularly the case due to the increasing prevalence of HIV infections in the study district.

### 3.3 Commonly used anti-malarial herbal remedies

A number of herbal remedies was mentioned as used in the management of malaria. Among these, remedies from three tree species, namely neem (*Azadirachta indica*), moringa *(Moringa oleifera*) and to a lesser extent jatropha (*Jatropha curcas*), were said to be frequently employed by most of the respondents.

“*When one of my household members is ill of malaria the first thing I do is to take leaves from the neem or moringa tree and prepare a remedy which I administer orally to the patient. Alternatively, I use jatropha seeds and prepare a remedy which I also administer orally to the patient”. (Man, GVH Sekeni 2)*

However, the use of jatropha is localised to one area of Chikwawa District (East Bank of the Shire River) where the trees are abundant. Also, its mode of preparation, which is laborious and time consuming, is discouraging its home use for the treatment of malaria. Therefore, the majority of the local communities resort to neem- or moringa-based remedies. This emerged when the respondents were asked to choose a treatment of preference given jatropha, neem, moringa and the modern anti-malarial drug Artemether-Lumefantrine (AL).

“*I will go for neem first. This is because neem is very effective against malaria and it acts quickly;; relieving the patient of pain and fever almost immediately. Jatropha, even though it is equally effective and fast acting its preparation takes time as the seeds need to be dried in the sun before use. With neem, if powder is not available I can just prepare a remedy from fresh leaves and administer it orally; within a few minutes the patient is cured. Modern treatment takes days for one to get cured”. (Woman, GVH Sekeni 2)*

### 3.4 Herbal combination treatments

Herbal combination treatments were reportedly prepared by mixing neem with other plants like moringa or *Aloe vera* for oral administration or mixing neem with mango, lemon and mpungabwe (*Ocimum americanum* L.) leaves for steam therapy. Because *Aloe vera* is not as common in the area as neem and moringa, community members preferred to use a neem-moringa preparation for prevention and treatment of malaria. Usually, this combination treatment involved a mixture of the plants at the ratio of about 2 parts of moringa to 1 part of neem and it was either pre-pared using powders or freshly extracted leaf fluids. The addition of moringa was said to help in reducing the bitterness of neem.

“*In case of illness of malaria, I mix 2 tablespoons of moringa powder with one tablespoon of neem powder. The mixed powders can either be dissolved in hot water or added to porridge and administered to the patient orally. After that I just observe to see how the patient is improving”. (Woman, GVH Misongwe)*

Because both plants are believed to possess anti-malarial properties, the majority of the respondents perceived that the combination of neem-moringa was able to boost the effectiveness of the herbal treatment, thus providing quick relief and fast recovery of the patient.

### 3.5 Choice of treatments according to age of the patient

The decision to resort to herbal remedies for the treatment of malaria as well as the type of herbal preparation and mode of administration was influenced by the age of the patient. Regarding the more frequently used neem preparations, the dose in children was reportedly based on half a tablespoon of leaf powder, essentially half the adult dose. However, it was reported that herbal remedies should not be administered to babies or very young children, given that children’s illnesses are difficult to be properly diagnosed at home.

“*Neem-based remedies can be orally administered to those who are 10 years and above without difficulties. Babies definitely cannot be given neem-based remedies;; we just take them straight to the hospital. For children under the age of five it is dangerous to give neem because at that age they cannot articulate well how they are feeling in their bodies and these remedies are very strong. So, if we ad-minister neem-based remedies to such ages we do it with fear even though we can properly make diagnosis. If I need to give neem-based remedies to children of about 2 years or above, I cook porridge first, add sugar and then I add a little bit of neem”. (Woman, GVH Sekeni 2)*

Apart from the risk of misdiagnosing their illness, it was also reported that young children are often taken to the hospital to avoid embarrassment when the child dies as the parents or caregivers may be thought of as being negligent. However, in situations where laboratory diagnosis of malaria is confirmed but no treatment regimen suitably de-signed for children is available, caregivers tend to still administer neem-based remedies either as boiled neem leaves supernatants or mixed with moringa to mask the bitterness. In case of infants who are still being breastfed a different formulation was reportedly being prepared as described below.

“*Last week I went to the hospital with my child who is 6 months old and she was diagnosed with malaria in their laboratory. But the doctor told me that they did not have the regimen suitable for her age and I was asked to go and buy it from a pharmacy. I did not have money to buy the drugs so I returned home and administered neem-moringa remedy. I boiled fresh neem and moringa leaves separately and thoroughly mixed the cooled down supernatant. Then I made an aperture on an egg and thoroughly mixed the remedy with the contents of the egg. I administered the mixture using the same egg as if she is breast-feeding. Within 3 days my child was OK” (Woman, GVH Sekeni 1)*

### 3.6 Choice of treatment according to seriousness of malaria illness

While it is common practice to administer antipyretics and analgesics followed by neem or other herbal remedies as first-line treatment for malaria, respondents reported that they endeavour to take the patients to the hospital as soon as the illness is noted to become serious in order not to risk the life of the person. However, in areas where free health care is not easily accessible, some members of the community reportedly use moringa for the management of severe malaria, particularly in cases where patients experience convulsions or seizures.

“*When the patient has convulsions or seizures we do not use neem because we know this type of malaria always follows bad spirits and it is those that cause convulsions. In that case we prepare a remedy of moringa, taking bark cuts and soaking them in water. When they are fully soaked we administer part of the remedy to the patient orally while we use the rest of it to bathe the patient. With-in a short time the patient gets well”. (Woman, FDG; GVH Sekeni 2)*

### 3.7 Effectiveness of neem remedies

When malaria signs and symptoms included gastrointesti-nal disturbances, respondents felt neem-based remedies were more effective than AL as they are perceived to work on both malaria infection and gastro-intestinal manifestations, such as diarrhoea and vomiting. Neem-based remedies were also believed to alleviate anaemia, which con-tributes to the preference of neem-based remedies.

“*In my case I also cut down the bark of neem tree facing the East and the West and boil them in a pot. The cooled down supernatant also helps to alleviate anaemia”. (Woman, FGD;; GVH Misongwe)*

Neem-based herbal remedies were perceived as effective by a large majority of the respondents, 97% of women and 77% of men held this opinion. When participants at FGDs were asked to take a ranking exercise (by show of hand, with eyes closed) of the effectiveness of AL and neem-based remedies for treating malaria, neem-based remedies did not show inferiority: 57% of men and 71% of women indicated that they perceived neem to be more effective than AL. Neem-based remedies were reportedly a preferred anti-malarial treatment by local communities be-cause it was perceived that its mode of action is fast and provides immediate relief unlike modern anti-malarial drugs. Thus, 98% of women and 52% of men confirmed to frequently use neem for the management of malaria.

### 3.8 Adverse events associated with neem remedies

All the respondents perceived that neem or neem-combination remedies were safe, stating that they have never noticed any side effects. However, they reported that neem-based remedies are contra-indicated during pregnancy, since they may induce miscarriage in pregnant women.

“…, *no pregnant woman is this community takes neem as treatment for malaria because they are aware of the con-sequences. When a pregnant woman takes neem-based remedy, its bitterness affects every organ in her body and this also affects the unborn child leading to miscarriage…” (Woman, GVH Misongwe)*

### 3.9 Moringa and neem as food and beverage supplements

Moringa and neem remedies were perceived to possess prophylactic properties that prevent malaria and other infectious diseases. For instance, it was reported that HIV-infected patients are encouraged by medical doctors to use moringa as food supplement because of its nutritional and medicinal properties.

“*My relative is HIV-infected, so at one point she was advised by doctors at the hospital that when she is home she should air-dry moringa leaves and ground them into powder. And then she should be using this powder on a daily basis by adding it to porridge or tea in order to boost her immune system. The same she was told to do with neem since this plant also acts as prophylaxis to opportunistic infections in those suffering from HIV/AIDS.” (Woman, FGD;; GVH Sande)*

Neem extracts are also used to fortify locally brewed beer. Brewers, usually women, believe that, besides the protective effects of neem against malaria and other diseases, neem-fortified beer also has a better taste, which attracts consumers to selling points where such beer is produced. Advantages of drinking neem-fortified beer were also re-ported by men themselves, confirming that neem supplementation would protect them against malaria infection, especially when they consume beer in excess and sleep without using mosquito nets. In addition, the majority of the respondents also perceived that consumers of neem-fortified beer do not suffer from the aftermath the following day. When triangulated with women brewers through IDI similar reasons for fortifying local beer with neem were pointed out.

“*Essentially, when I am adding neem to beer I know consumers will also be protected from malaria. However, I add neem to beer not only because of malaria, but because it works against so many illnesses;; malaria is just one of them. And again neem improves the body’s immune response to the benefit of those who are immuno-compromised by infections such as HIV”. (Woman, beer brewer;; GVH Sande)*

Neem food supplements were also reportedly liked be-cause of their capacity to improve sexual drive, especially in men. This was ascertained by both men and women respondents.

### 3.10 Locally prepared neem and moringa products

Beside neem-fortified beer, leaf powder preparations of neem and moringa are locally produced and sold in markets both in urban and rural communities. The majority of the interviewees expressed interest in having various consumables fortified with neem, moringa or a combination of both, as they felt this would serve the population who could benefit from the medicinal effects of neem and moringa as well as from the nutritional value of moringa. The suggested products ranged from supplemented juices, cooking oil, tea leaves and powder for porridge.

The respondents also felt that if neem can be encapsulated and made accessible, the product could help, especially during the high transmission season, to protect community members against malaria. Interestingly, some people were already encapsulating neem powder to mask its bitterness for the consumer. Encapsulating neem leaf powder was probably the most important aspect, as, at local level, the majority of the respondents felt that the efficacy of neem against illness is contained in its bitterness and therefore encasing neem in capsules or coating for taste masking would ensure that the product is palatable while the perceived effective powers of the bitterness is retained.

## 4 Discussion

This study identified neem and moringa as the two principal plants used in the management of malaria at community level in the Lower Shire Districts of Chikwawa, Malawi. Various preparations of these plants, based on one or both of these tree species formulated as combination remedies, are employed at household level to cure or prevent malaria. Moringa- and neem-based remedies are widely used as first-line home-based remedies. Factors influencing this treatment behaviour include its perceived effectiveness and its fast action against the clinical signs of the disease. Lack of proper medical attention in modern health facilities might probably have contributed to the preference for herbal home-based malaria management. Neem-and moringa-based remedies were perceived safe and were readily given to adults and children. However, restricted application was reported in very young children, and neem -based remedies were perceived to cause adverse events in pregnant women.

Local communities widely use herbal remedies from neem, moringa or a combination of both as first line treatment for malaria. Essentially, as soon as signs and symptoms of malaria are noticed, antipyretics and analgesics were reportedly administered to the patient. These drugs were either leftovers from previous treatments sought from medical facilities or drugs that were bought in local shops. Administration of antipyretics to patients with fever before presentation for treatment is common in various African countries. Recent studies in Tanzania indicated that over 76% of patients with febrile illness had antipyretics administered at home before seeking medical care [19]. If illness persists, caregivers then resorted to home treatment with neem- and moringa-based remedies. Usually, malaria illness was reportedly resolved after administration of these herbal preparations. However, if illness persisted, the patient was rushed to a health facility for proper diagnosis and treatment, as it is believed that treatment failure fol-lowing administration of neem-based remedies would result from other illnesses.

In our study area, neem and moringa remedies are widely used as anti-malarial prophylaxis. However, the bitterness of neem remains a challenge for regular consumption. Nevertheless, at community level, it is believed that the effective power of neem against infections is contained in its bitterness. It was on this basis that some members of the community encapsulate neem powder in order to offset the bitterness while not interfering with the perceived effective bitter components. There is some scientific evidence suggesting that the principle bitter element of neem, nimbidin, possesses anti-pyretic activities [20].

The use of neem-combination herbal remedies goes beyond the management of malaria. They are also used for the prevention of other illnesses like diarrhoea and sexually transmitted infections, including HIV. While the use of neem extracts as anti-malarial remedies is well documented (reviewed in [21,22]), evidence is now emerging that fractionated neem extracts possess antiretroviral proper-ties, which appear to interfere with invasion of the virus into lymphocytes and boost CD4+ cell counts [23]. A fractionated neem leaf preparation administered to HIV-infected patients was found to increase mean lymphocyte counts, haemoglobin concentration and mean body weight, and it was noted to be well tolerated [24]. Given this evidence and the high prevalence (30%) of HIV infections in our study district, which is almost twice the national aver-age [25], the use of neem-based remedies by HIV-positive individuals might improve their clinical situation and provide some protection against co-infection with malaria and other infectious agents.

Treatment-seeking choices of patients and caregivers regarding the management of malaria were not influenced by the geographical accessibility of free health care provided by government facilities. This is in contrast to observations from other studies, where it was found that distance to the nearest health facility and related costs and travel time was found to impact on utilisation of health care facilities [26]. Our data demonstrated that members of the local communities in Chikwawa use herbal remedies, particularly neem and moringa leaves, as first-line treatment for malaria due to the erratic supply of anti-malarial drugs at both the free governmental health facilities and private health facilities.

From our FGD and IDI, it emerged that women were most frequently the first to diagnose symptoms of illnesses at the household level. For common signs and symptoms of illness, women were reported (by both women and men) to be in a better position to assess the condition of the patient and administer the appropriate home-based remedies on their own. At community level, men and women seemed to have a comparable breadth of knowledge regarding the most commonly used home-based remedies. However, women generally tended to know more about the various modes of preparations than their male counter-parts. This might be due to the experience of regularly collecting, preparing and administering the remedies to children and adult family members.

While a large proportion of women preferred neem-based remedies as first-line treatment for malaria due to its perceived fast activity against illness, the abundance of neem and moringa trees around the communities and ease of preparation of the remedies also might have played a role in this preference. Women, who most of the time are fully charged with taking care of the household, were also reportedly engaged in small-scale business to contribute to the household welfare. As a result, they are likely to prefer a treatment regimen which is easy to obtain and which also resolves the illness as quickly as possible so that they do not lose time for money generating activities.

Our findings highlighted the perception of efficacy and safety of neem, moringa or neem-moringa combination herbal remedies by members of the local communities. Herbal remedy users perceived that in the management of malaria, neem was not inferior to the current anti-malaria regimen;; in fact, the majority believed that neem-based remedies were more efficacious than AL, as they are fast-acting and therefore provide cure in a shorter period of time. Based on our findings and the high prevalence of use of neem, moringa or neem-moringa combinations, our data showed the social acceptance of these herbal products at community level. While the perceived effectiveness and safety of these herbal remedies might have been one of the determining factors for such high acceptability, encouragement by medical doctors for HIV-infected patients to use moringa herbal remedies in the management of their illnesses might also have contributed to this acceptance.

The main limitation of this study (restricted to four communities) is that it remains unclear whether its findings are broadly applicable. Focus group discussions and in-depth interviews are costly and time-consuming;; there-fore, a few sets of objectives were planned in order to en-sure that the aims of the study were achievable within a limited time and with budget constraints. Saturation of ideas was achieved after interviewing a relatively low number of participants, who cannot be considered representative of the entire Chikwawa District. Furthermore, purposively sampling for in-depth interviews meant that only individuals who were considered to have breadth of knowledge on herbal remedies might have provided rich information;; however, what they reported might equally not be representative for the communities they were sampled from.

Despite these limitations, critical baseline information on the use of neem-based remedies for the management of malaria was obtained in this study. It underscores the need to conduct larger qualitative as well as quantitative studies to determine not only the magnitude of use but also evaluate clinical efficacy and safety of these remedies in consumers. This would help to provide direction for the development of standardised phytomedicines as first-aid treatments or of improved neem-based formulations, which may be used for mass administration during high transmission seasons in order to reduce malaria cases and transmission intensity at community level. Efforts for the development and production of such herbal medicines need to ensure involvement of local communities to encourage cultural synthesis and therefore guarantee acceptance and sustainability at community level.

## 5 Conclusions

Given the high utilisation of neem and/or moringa combination remedies, their perceived ease of access, preparation and administration without frequent adverse events as well as their perceived effectiveness and safety, there is a possibility that standardised herbal products made from these tree species would be acceptable for mass administration as anti-malarial prophylaxis. However, the perceived adverse events of neem-based remedies in pregnancy present a major drawback and may hamper efforts to fortify products needed for general consumption.

Members of the local community, who frequently use herbs for their primary healthcare needs, perceived that home-based remedies were efficacious in the management of malaria. In some instances, neem-based remedies were perceived to be more efficacious than modern antimalarial medicines owing to their potential to act very fast and providing immediate relief to the patient. We suggest that this perception of herbal remedies efficacy might be a major contributing factor influencing increase in use of herbs, particularly neem-based remedies, in the management of malaria. Furthermore, erratic supply of antimalarial drugs in modern health facilities might have sustained this use. Evidence-based research is required to validate parasitological and clinical efficacy and determine safety for directing populations towards a proper use of these herbal remedies.
